# A Zebra Diagnosis: A Case of Spontaneous Hematomyelia Associated With COVID-19

**DOI:** 10.7759/cureus.40452

**Published:** 2023-06-15

**Authors:** Elham Ahmed, Kimia Saleh-Anaraki, Chima Onyegbule, Mamoon AlAfun, Hussam M Ammar

**Affiliations:** 1 Internal Medicine, University of Maryland Capital Region Medical Center, Largo, USA

**Keywords:** covid-19, laminectomy, spinal hemorrhage, paralysis, spontaneous spinal hematomyelia

## Abstract

Spontaneous non-traumatic spinal hematomyelia, characterized by intramedullary spinal hematoma, is a rare neurological emergency. Bleeding arteriovenous malformation, coagulopathies, and neoplasms are reported causes of this rare diagnosis. The authors present a case of a previously healthy man who presented with acute paraplegia and was found to have a spontaneous hematomyelia in association with covid infection. He underwent laminectomy and hematoma evacuation but did not recover any neurological function.

## Introduction

Spinal cord hemorrhage is divided based on the etiology into traumatic or non-traumatic "spontaneous." It is categorized based on the anatomic compartment affected: intramedullary "hematomyelia," subarachnoid, subdural, or epidural. The most common etiology is trauma, followed by spinal arteriovenous malformation. Spontaneous epidural hemorrhage has an incidence of 0.1 per 100000, and it is four times more common than subdural hemorrhage. The exact incidence of other types of spinal hemorrhage isn't precisely documented in the literature [1-4].

## Case presentation

**Figure 1:**
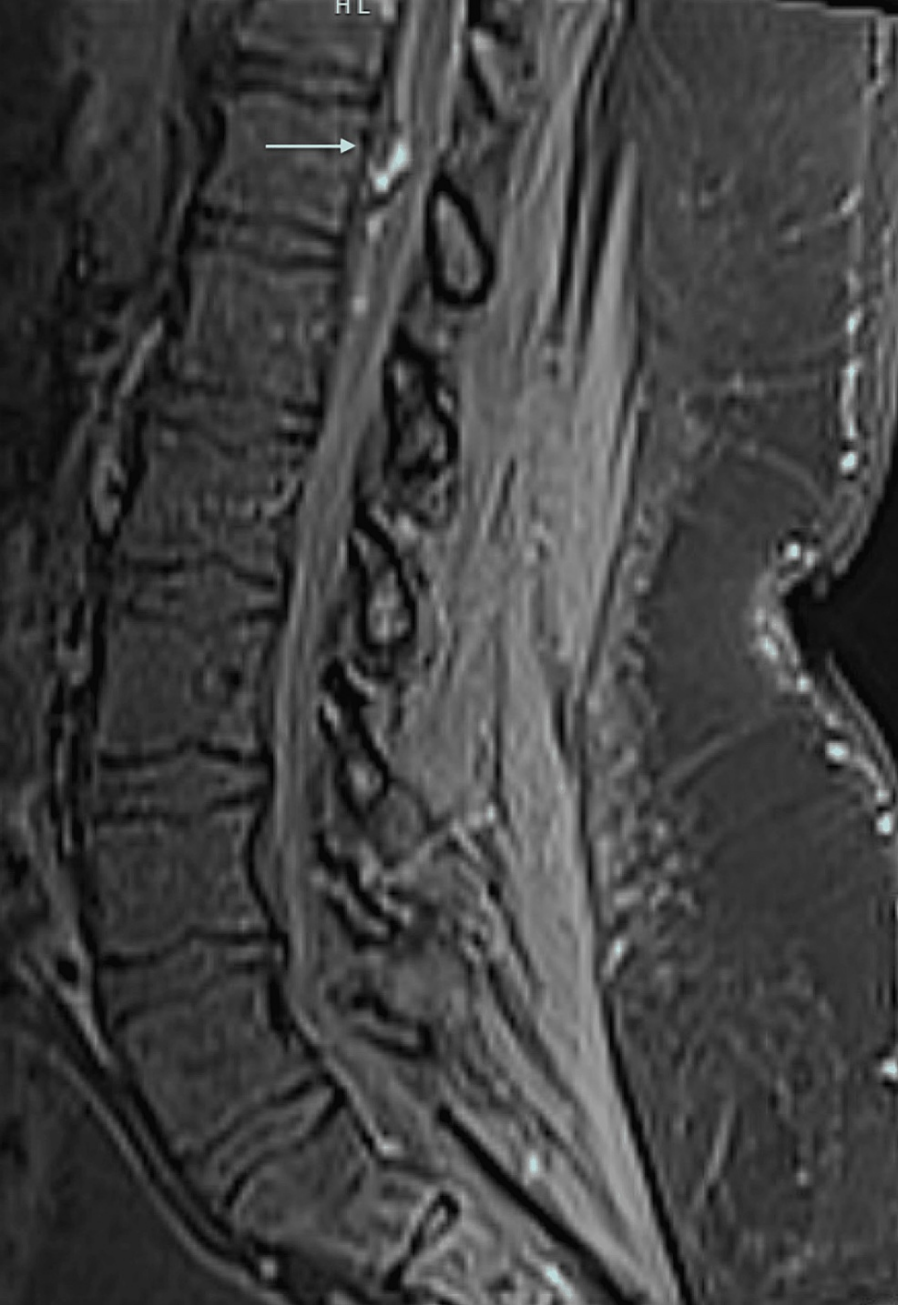
Preoperative Magnetic Resonance Imaging of the Spinal Cord Non-homogeneous intramedullary extramedullary lesion measuring 1.3 cm at T12.

**Figure 2:**
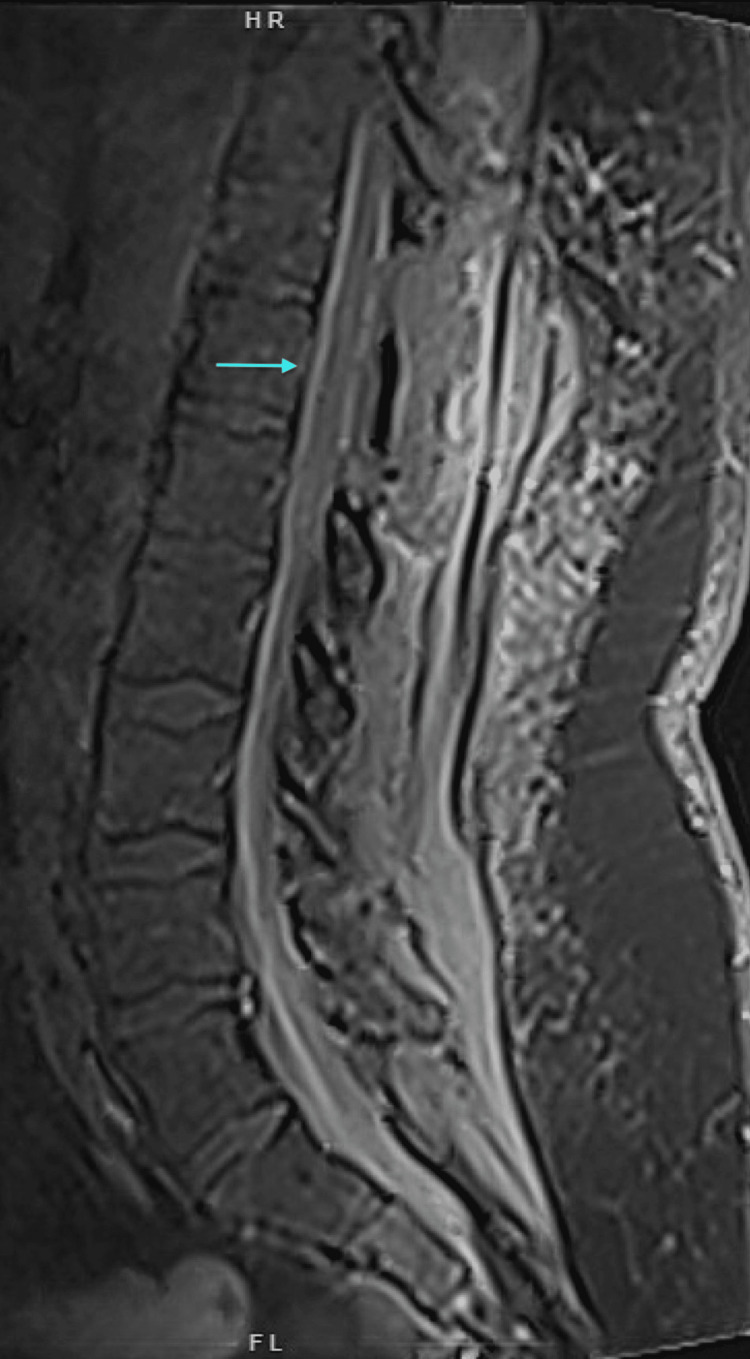
Postoperative Magnetic Resonance Imaging of Spinal Cord Evacuation of the hematoma and postoperative changes after laminectomy.

A previously healthy 51-year-old man presented to the emergency department with acute bilateral leg weakness, lower back pain, and urine retention. Four days before this presentation, he experienced a sore throat, runny nose, subjective fever, and cough. Two days before this presentation, he was diagnosed with mild COVID-19 infection. He started to develop bilateral progressive leg weakness to the extent that he could not stand on his feet when he called the emergency medical service. He was not taking any medications at the time of presentation except for acetaminophen. Upon admission, the patient's vital signs were a blood pressure of 207/143 mmHg, temperature of 36.7°C, respiratory rate of 16/min, and pulse rate of 90/min. Motor strength was 1/5 proximally and 2/5 distally in the left lower extremity and 2/5 proximally and 3/5 distally in the right lower extremity. Upper extremities strength, reflexes, and sensation were normal. Deep tendon reflexes were absent, and the touch and proprioception sensations were intact. Laboratory results showed white blood cells at 10.2×10^9/L (reference 4.5-11.0×10^9/L), hemoglobin at 15.8 g/dL (reference 13.5-17.5g/dL), platelet counts at 387×10^9/L (reference 150-350×10^9/L), sodium at 139 mmol/L (reference 136-145mmol/L), potassium at 3.7 mmol/L (reference 3.5-5.0 mmol/L), creatinine at 1 mg/d (reference <1.5ng/dL), international normalized ratio (INR) at 1, activated partial thromboplastin time (APTT) at 26 seconds (reference 22.1-35.1 second), and D-Dimer (age-corrected) was elevated at 1.5 mg/L (reference <0.5 mg/L). Chest radiography was unremarkable. Magnetic resonance imaging (MRI) of the spine revealed a non-homogeneous intramedullary and extramedullary lesion measuring 1.3 cm at T12 (Figure 1). The patient was admitted to the intensive care unit and received intravenous dexamethasone 10 mg intravenous every 6 hours for six days, and it was tapered and discontinued after 12 days. A decompressive laminectomy and evacuation of the intramedullary and subdural hematoma were performed; no arteriovenous malformation was found during surgery (Figure 2). Postoperatively, the patient's motor strength worsened, with zero strength in the left lower extremity and only 1/5 strength in the right hip flexion. The patient was subsequently transferred to a rehabilitation facility but did not experience any improvement in neurological function after four weeks of rehabilitation.

## Discussion

Intramedullary spinal cord hemorrhage was first reported in 1814 [2]. The term hematomyelia was introduced in 1827. Depending on the level of the lesion, the patient may present with paraplegia or quadriplegia, bowel or bladder dysfunction, and sensory disturbances. Hematomyelia usually presents with rapid progressive myelopathy, as in our case. Although spontaneous hematomyelia in the absence of tumors, vascular malformations, coagulopathies, or syringomyelia has been reported, it is exceedingly rare [2,4]. COVID-19 has been associated with various spinal cord complications, including myelitis, Guillain-Barre syndrome, encephalomyelitis, and spinal cord ischemia secondary to spinal artery thrombosis [5,6]. In our patient, the initial working diagnosis was myelitis; however, MRI revealed a spinal mass, and the final diagnosis was not confirmed until surgery was performed and a pathological exam confirmed hematoma. In the medical literature, spinal cord hemorrhage has been reported twice in patients with COVID-19 infection (Table 1). Sabouri et al. reported a case of 73 year old who was admitted with respiratory failure secondary to COVID-19 pneumonia. He developed extremities weakness on day 20 and subsequently was diagnosed with hematomyelia. He had undergone a laminectomy and evacuation of the hematoma with the improvement of his weakness [7]. Dlake et al. reported a 72-year-old woman who presented with mild covid symptoms, severe back pain, and instability. She was found to have a spinal subdural hematoma. She had some recovery of her leg weakness after the laminectomy [8].

**Table 1:** Cases of Spinal Hemorrhage Associated with COVID-19 MRI: magnetic resonance imaging

Case	Primary presentation	Time of neurological deficits	Neurological deficits	Anticoagulation	MRI findings	Surgery	Type of spinal hemorrhage	Prognosis
Sabouri et al., 2022 [7]	Respiratory failure, covid pneumonia	Day 20 of hospitalization	Weakness of upper and lower extremities, decreased sensation, and absent tendon reflexes. Rapidly progressed to paraplegia, neurogenic bladder, and bowel.	Heparin 5000 units subcutaneously every 12 hours	Mixed intensity extending from C3 to T3	Laminectomy and evacuation of the hematoma	Intramedullary	Partial improvement of his arm and leg weakness.
Dlaka et al., 2022 [8]	Upper back pain, instability, and mild covid symptoms	On the day of admission	Left leg weakness followed by right leg weakness. This progressed to left leg paralysis, neurogenic bowels, and bladder.	None	Subdural hematoma extending from C7 to T3 spinal cord signal suggestive of myelopathy	Laminectomy and evacuation of the hematoma	Subdural	Partial Improvement of his leg weakness

COVID-19 has been associated with both thrombosis and hemorrhage. Thrombosis is the most common phenotype of COVID-19 infection. The overall venous thromboembolism rate is 12% to 31% in COVID-19 inpatients [9]. Hemorrhage is the less common phenotype of COVID-19. A systematic review of > 18000 patients had a pooled incidence of 7.8% for minor bleeding and 3.9% for life-threatening bleeding. The highest incidence of bleeding was reported in patients on intermediate and high-dose anticoagulation (21.4%) [10]. A retrospective study of 400 patients who were receiving standard dose prophylactic anticoagulation had a minor bleeding rate of 4.8% and a life-threatening bleeding rate of 2.3% [11]. Bleeding complications have been reported in various presentations, including central nervous system hemorrhage, gastrointestinal bleeding, muscular hematoma, hemoptysis, and renal hematoma [9,11]. Elevated D-Dimer was associated with both thromboses and bleeding in this study, while disseminated intravascular coagulopathy, clinically relevant thrombocytopenia, and reduced fibrinogen were rare and were associated with bleeding complications [11]. Blood-brain barrier and endothelial injury secondary to hypoxia and inflammatory response might cause microbleeds and eventually brain and spinal cord hemorrhage, particularly in the presence of anticoagulation use [7,8,11]. The association of COVID-19 with hematomyelia in the current literature cannot prove causation.

## Conclusions

Spinal cord hemorrhage associated with COVID-19 is an extremely rare complication. It can happen spontaneously without trauma, coagulopathy, or arteriovenous malformation. Recognizing this association and intervening early is crucial to preventing permanent neurological deficits. Physicians were taught to think of horses, not zebras when they hear hoofbeats. Think zebras; rare diagnoses exist, and creating a broad differential diagnosis can prevent you from having a wrong cognitive turn.
